# Optimized Model Predictive Controller Using Multi-Objective Whale Optimization Algorithm for Urban Rail Train Tracking Control

**DOI:** 10.3390/biomimetics11010060

**Published:** 2026-01-10

**Authors:** Longda Wang, Lijie Wang, Yan Chen

**Affiliations:** 1 School of Electrical Engineering, Dalian Jiaotong University, Dalian 116028, China; 2 School of Mechanical and Electrical Engineering, Chizhou University, Chizhou 247000, China

**Keywords:** urban rail train, tracking control, multi-objective whale optimization algorithm, model predictive controller, control parameters

## Abstract

With the rapid development of urban rail transit, train operation control is required to meet increasingly stringent demands in terms of energy consumption, comfort, punctuality, and precise stopping. The optimization and tracking control of speed profiles are two critical issues in ensuring the performance of automatic train operation systems. However, conventional model predictive control (MPC) methods are highly dependent on parameter settings and show limited adaptability, while heuristic optimization approaches such as the whale optimization algorithm (WOA) often suffer from premature convergence and insufficient robustness. To overcome these limitations, this study proposes an optimized model predictive controller using the multi-objective whale optimization algorithm (MPC-MOWOA) for urban rail train tracking control. In the improved optimization algorithm, a nonlinear convergence mechanism and the Tchebycheff decomposition method are introduced to enhance convergence accuracy and population diversity, which enables effective optimization of the initial parameters of the MPC. During real-time operation, the MPC is further enhanced by integrating a fuzzy satisfaction function that adaptively adjusts the softening factor. In addition, the control coefficients are corrected online according to the speed error and its rate of change, thereby improving adaptability of the control system. Taking the section from Lvshun New Port to Tieshan Town on Dalian Metro Line 12 as the study case, the proposed control algorithm was deployed on a TMS320F28335 embedded processor platform, and hardware-in-the-loop simulation experiments (HILSEs) were conducted under the same simulation environment, a unified train dynamic model, consistent operating conditions, and an identical evaluation index system. The results indicate that, compared with the Fuzzy-PID control method, the proposed control strategy reduces the integral of time-weighted absolute error nearly by 39.6% and decreases energy consumption nearly by 5.9%, while punctuality, stopping accuracy, and comfort are improved nearly by 33.2%, 12.4%, and 7.1%, respectively. These results not only verify the superior performance of the proposed MPC-MOWOA, but also demonstrate its capability for real-time implementation on embedded processors, thereby overcoming the limitations of purely MATLAB-based offline simulations and exhibiting strong potential for practical engineering applications in urban rail transit.

## 1. Introduction

Urban rail transit systems are distinguished by their efficient, low-carbon operation and high passenger capacity, and have become a core component and essential infrastructure of modern urban transportation networks. They serve as a key pathway to achieving low-carbon urban mobility and smart travel [1]. With the continuous advancement of automation and intelligence, passengers place increasing demands on the safety and comfort of train operations. As the core of automatic train operation systems, the optimization and precise tracking of speed trajectories directly determine overall operational performance. Although a variety of efficient speed trajectory optimization methods have been proposed in recent years, relying solely on the optimal speed profile is insufficient to ensure performance. High-precision trajectory tracking control strategies are also required to guarantee stable execution of the scheduled speed [2]. At present, PID control remains one of the most commonly used control strategies in urban rail train operation control systems. Owing to its error-feedback mechanism, PID control features a simple structure and ease of implementation, and has therefore been widely adopted in engineering practice [3]. However, conventional PID control lacks the capability to predict future system states, making it difficult to effectively cope with nonlinear characteristics and external disturbances under complex operating conditions. As a result, limitations persist in terms of speed trajectory tracking accuracy, energy consumption control, and passenger comfort [4]. To enhance control performance, recent studies have increasingly combined PID control with artificial intelligence techniques and meta-heuristic optimization algorithms [5], such as particle swarm optimization (PSO) and genetic algorithms (GA), to achieve intelligent tuning of controller parameters [6]. Although these approaches improve the robustness of PID control to some extent, their primary focus remains on parameter optimization. Consequently, they do not fundamentally address the lack of predictive capability in the control strategy, leaving further room for improvement in high-precision trajectory tracking under complex operating conditions. Therefore, the development of more efficient and robust control strategies that can maintain high-precision trajectory tracking under complex operating conditions is of great significance for improving the performance of urban rail transit systems.

The trajectory tracking control problem of urban rail trains is, in essence, a typical issue of optimal control and stability control. Such problems are also common in other domains. For example, Hentout et al. proposed a strategy combining shortest-path planning with fuzzy logic control to achieve efficient path tracking of mobile robots in both static and dynamic indoor environments [7]. Wen et al. developed an improved cascade control method for the single-point levitation system of maglev trains to ensure stable levitation [8]. Chai et al. presented an optimal trajectory tracking guidance approach based on receding horizon control for spacecraft reconnaissance missions, ensuring flight stability under complex dynamic conditions [9]. These studies indicate that, whether in robotic navigation, maglev systems, or spacecraft trajectory control, optimization and stability are consistently central objectives in control design. Accordingly, speed tracking of urban rail train under complex operating conditions must simultaneously consider operational efficiency and system stability.

Consequently, researchers have introduced methods such as optimal control and robust control into train operation control to compensate for the limitations of PID control. However, these approaches often rely heavily on precise mathematical modeling of the train system, a requirement that is difficult to fully satisfy given the complex and variable operational environment of urban rail transit [10]. In recent years, to further enhance the trajectory tracking accuracy and stability of automatic train operation systems, various advanced control methods have been proposed and investigated, including adaptive control, sliding mode control (SMC), iterative learning control (ILC), active disturbance rejection control (ADRC), and model predictive control (MPC). Specifically, Huang et al. proposed a parking control method based on robust adaptive backstepping, which significantly improved train stopping accuracy, though it imposes a high computational burden and still suffers from limited real-time performance under complex conditions [11]. Kang et al. designed a speed-tracking controller based on fuzzy SMC, effectively enhancing system robustness and tracking performance, but it is highly dependent on parameter tuning [12]. In addition, Liu et al. employed a multi-mass dynamic model to develop adaptive ILC, achieving precise tracking under velocity constraints; however, the high algorithmic complexity limits its real-time applicability [13]. Wang et al. incorporated the artificial bee colony algorithm into ADRC design to improve disturbance rejection performance, though the convergence speed of the algorithm remains a concern [14]. Overall, while these methods have advanced trajectory tracking and disturbance rejection performance, they generally suffer from limited real-time capability and strong parameter dependence. In contrast, MPC, with its superior multi-constraint handling and receding-horizon optimization features, can achieve higher accuracy and stability in trajectory tracking under complex operating conditions. For instance, Felez et al. applied MPC to train virtual coupling systems, significantly improving the coordinated control of multiple train speed trajectories [15], while Novak et al. proposed an energy-efficiency-prioritized MPC method, which effectively reduced traction energy consumption while maintaining smooth velocity profiles [16]. Therefore, owing to its outstanding performance, MPC has gradually become a research focus in the field of urban rail train tracking control. A comparison of urban rail train control methods is provided in Table 1.

MPC was initially widely applied in process industries and, owing to its advantages in receding-horizon optimization and multi-constraint handling, has gradually been extended to domains such as motor control, energy management, and vehicle control [17]. In the trajectory control of urban rail train, MPC predicts future operational states based on the train dynamic model and optimizes traction and braking commands in real time at each sampling interval, thereby achieving high-precision speed tracking. However, conventional MPC in complex operating environments, such as frequently varying gradients and curves, or open coastal lines-relies heavily on model accuracy and parameter settings, which can limit control performance. To address these limitations, various improved MPC (IMPC) methods have been proposed. For example, Zhou et al. developed distributed MPC (DMPC) to achieve coordinated local and global tracking, though its adaptability and accuracy remain limited [18]. Chen et al. designed hierarchical optimization MPC (HO-MPC), which performs well in trajectory planning and scheduling but depends on accurate models and lacks adaptive capability [19]. To enhance stability, Wang et al. combined fuzzy logic with robust predictive control (RFPC) to improve tracking accuracy on high-frequency lines, but real-time adaptability is still constrained [20]. Additionally, Gao et al. employed a Koopman-based model to develop collaborative braking MPC (CB-MPC), enhancing multi-train consistency control, although its generalization capability is limited [21]. Chen et al. proposed multi-objective parameter-tuning MPC (MOT-MPC), which improves responsiveness and stability but relies on offline parameter adjustment and lacks flexibility for dynamic environments [22]. Overall, IMPC has partially improved trajectory tracking performance, but it remains constrained by model dependency and parameter sensitivity.

Building on this foundation, researchers have recently attempted to integrate intelligent optimization algorithms into the MPC framework to further enhance its adaptability and global optimization capability in complex trajectory tasks [23]. For example, Nobahari and Nasrollahi embedded ant colony optimization (ACO) into nonlinear MPC to improve robustness and accuracy; however, the method is highly sensitive to parameter settings, making it less suitable for frequently changing train operating environments [24]. Diwan and Deshpande proposed a fast MPC approach combining parallel PSO with a divide-and-conquer strategy, which increases computational efficiency but still faces trade-offs between convergence speed and accuracy in multi-objective coordination [25]. Lin et al. designed a multi-objective PSO strategy for virtually coupled systems to achieve a compromise among energy consumption, safety, and tracking precision, yet its applicability to general trajectory tracking scenarios is limited [26]. Furthermore, Wang et al. employed wavelet neural networks to model nonlinear dynamics and integrated them with MPC; although this improves nonlinear response, the approach suffers from complex model training and limited robustness [27]. Among existing metaheuristic optimization algorithms, the Whale Optimization Algorithm (WOA) has attracted increasing attention in recent years due to its simple structure, limited number of control parameters, and its ability to maintain a favorable balance between global exploration and local exploitation [28]. These characteristics make WOA particularly suitable for integration into MPC frameworks, especially for parameter tuning problems that impose stringent requirements on real-time performance and constraint handling. Compared with GA and PSO, WOA exhibits lower dependence on parameter tuning and demonstrates better scalability when applied to complex control structures. Nevertheless, the standard WOA still inevitably suffers from issues such as premature convergence and degradation of population diversity in multi-objective optimization problems, which motivates further improvements to the algorithm and its deep integration with MPC in this study.

Biomimetics seeks to distill generalizable structural characteristics and behavioral principles from biological systems and transfer them into engineering-oriented frameworks for modeling, optimization, and control. In recent years, swarm intelligence optimization algorithms inspired by collective behaviors observed in nature have attracted growing attention, owing to their strong global search capability, adaptive behavior, and robustness when addressing complex nonlinear, multi-constraint, and multi-objective engineering problems. Representative approaches, such as PSO, ACO, and theWOA, emulate information exchange, cooperative search, and environmental adaptation among biological individuals, thereby enabling efficient solutions to challenging optimization tasks and providing an effective methodological bridge between natural intelligence and engineering control applications.

Although previous studies have made some progress in MPC parameter optimization and the application of metaheuristic algorithms, most existing work still focuses on single-objective optimization or multi-objective frameworks with relatively weak coupling between objectives. At the same time, no prior research has systematically incorporated the Tchebycheff decomposition method into the WOA for multi-objective tuning of MPC parameters in urban rail transit systems.

From the biomimetics perspective, the WOA is derived from an abstract representation of the cooperative bubble-net feeding behavior of humpback whales. By combining prey encircling, spiral updating, and stochastic search mechanisms, the algorithm achieves a dynamic balance between global exploration and local exploitation, reflecting the adaptive decision-making characteristics of biological swarms in complex environments [29]. The optimization process constructed on this behavioral principle exhibits strong capabilities in multi-objective optimization and constraint handling, enabling effective coordination among competing performance indices. As a result, it is particularly well suited for multi-objective tuning of MPC parameters in urban rail train trajectory tracking applications.

**Figure 1:**
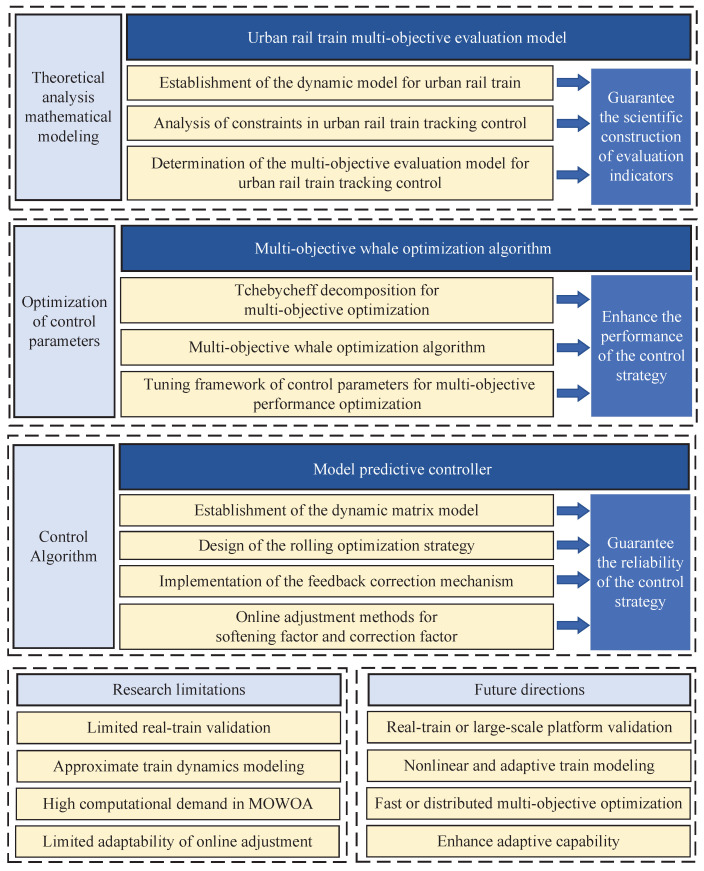
Research technical roadmap for this study.

This paper proposes a model predictive controller with multi-objective whale optimization algorithm (MPC-MOWOA). Based on a multi-objective evaluation model constructed for urban rail train tracking control, the MOWOA is introduced to effectively solve the challenge of control parameter tuning. Furthermore, a MPC is developed based on an online adjustment mechanism, which incorporates a softening factor and a correction coefficient. The effectiveness of the proposed method in enhancing the trajectory tracking precision of urban rail train is verified through simulation results. The comprehensive framework underlying this research is depicted in Figure 1.

The main contributions are summarized as follows:

(I) This study proposes a multi-objective comprehensive evaluation framework for urban rail train tracking control. The proposed framework evaluates tracking control performance from multiple dimensions, effectively addressing the limitations of conventional methods that rely on a single performance metric to assess control effectiveness. By jointly considering tracking accuracy, comfort, energy consumption, and punctuality, the evaluation framework enables a more comprehensive and objective assessment of the practical performance of different tracking control strategies. As a result, it provides a unified and quantitative basis for performance comparison and control parameter optimization.

(II) This study proposes the MOWOA. By introducing a nonlinear convergence factor and an adaptive weighting mechanism, the global search capability is enhanced, enabling the tuning of the controller’s initial parameters and thereby significantly improving control performance. Meanwhile, improvements are also made to the MPC: a softening factor with online adjustment based on fuzzy satisfaction indices is introduced to dynamically balance constraint satisfaction and performance enhancement; in addition, an adaptive correction coefficient strategy is proposed, which ensures system robustness while accelerating the response speed.

(III) This study conducts hardware-in-the-loop simulation experiments (HILSEs) to validate the effectiveness of the proposed approach in real-world operating environments. The experimental results demonstrate that, compared with traditional MPC and other control methods, the proposed strategy achieves superior performance in terms of comfort, energy consumption, and tracking accuracy. Furthermore, this study, for the first time, integrates the improved multi-objective optimization algorithm with MPC for urban rail train tracking control, thereby verifying the effectiveness and superiority of the method under complex operating conditions.

The structure of the paper is arranged as follows. Section 2 introduces the multi-objective evaluation model. Section 3 presents the MOWOA for optimizing MPC control parameters. Section 4 presents the MPC incorporating the online adjustment mechanism combining a softening factor and a correction coefficient. Section 5 presents the simulation experiments and corresponding analysis. Finally, Section 6 summarizes the conclusions of this study.

## 2. Multi-Objective Evaluation Model for Urban Rail Train Tracking Control

### 2.1. Train Dynamics Model and Assumptions

To implement urban rail train tracking control, the system must adhere to the dynamic equations of train operation outlined below. These equations describe the longitudinal motion of the urban rail trains, illustrating how position, velocity, and the acting force are interconnected in the direction of travel. The model serves as the basis for subsequent control algorithm design and trajectory optimization. The system dynamics are given as:

(1) $$\left\{ \begin{array}{l} \frac{dx}{dt}=V\\ m_{1}V\frac{dV}{dx}=F\left(u,V\right)-W\left(x,V\right) \end{array}\right.$$

where *x* represents the current location of the urban rail trains; *t* represents the real-time operation duration of the train; and *V* is the exact running velocity of the train; F(u,V) represents the force generated by the train; depending on the operating condition, it may act as either a driving or braking force; W(x,V) represents the total resistance experienced by the train, including both the basic resistance and additional resistance, expressed as $W\left(x,V\right)=r_{0}\left(V\right)+R_{e}\left(x\right)$. Here, $r_{0}\left(V\right)$ represents the basic resistance at the current train speed, while $R_{e}\left(x\right)$ represents the additional resistance at the current position, which accounts for external disturbances affecting train operation, such as gradient variations, aerodynamic fluctuations, and environmental factors.

(2) $$\begin{array}{c} m_{1}=\left(1+y\right)\times m_{0} \end{array}$$

where $m_{1}$ denotes the mass reflecting inertia of the urban rail train, *y* denotes the slewing mass factor of the train, and $m_{0}$ denotes the real mass of the train.

The function F(u,V) represents the interaction among the control input *u*, the train’s operating speed *V*, and its dynamic characteristics. Under traction mode, it reflects the traction performance of the train, whereas under braking mode, it characterizes the deceleration behavior. Here, *u* represents the control command applied to regulate the driving or braking system of the urban rail train [30].

### 2.2. Train Model

The train model serves as the foundation for automatic train operation and speed trajectory tracking control. However, due to system complexity and the coupled effects of multiple factors such as traction, braking, and resistance, it is challenging to obtain an accurate train dynamic model. The traction system of an urban rail train primarily consists of a control unit, braking resistors, and power converters; therefore, the train cannot be simply approximated as a single-point mass model. In practice, the traction force is generated by traction motors according to notch control commands, and the longitudinal motion of the train is realized under the combined influence of traction force and various running resistances.

**Assumption** **1.** *The train longitudinal dynamics can be approximated as a linear and time-invariant system within the studied time domain. Nonlinear characteristics, such as traction and resistance, are simplified through equivalent modeling or parameter linearization. Moreover, the collected operational experimental data are sufficient to represent the typical dynamic behavior of the system, making them suitable for system identification.*

On the basis of Assumption 1, and using operational experimental data, the system is identified by the least squares method, yielding a second-order transfer function model of train longitudinal motion. Its general form can be expressed as follows:

(3) $$G\left(s\right)=G_{1}\left(s\right)G_{2}\left(s\right)=\frac{612}{s+0.34}\cdot \frac{1}{8621s+822.4}=\frac{0.07128}{s^{2}+0.4356s+0.0324}$$

where $G_{1}\left(s\right)=\frac{612}{s+0.34}$ represents a fixed component corresponding to the train’s internal response, reflecting the intrinsic characteristics of the train that relate the traction level to the generated control force; $G_{2}\left(s\right)=\frac{1}{8621s+822.4}$ represents a variable component corresponding to the train’s external response, capturing the extrinsic characteristics that describe the relationship between the control force and the train’s operational speed and position.

After establishing the train model, it is necessary to discretize the continuous time transfer function, since the model predictive controller requires a discrete-time representation. In this study, the zero-order hold (ZOH) method was adopted to convert the continuous transfer function into a discrete model with the specified sampling period, ensuring consistency between the plant model and the controller in the discrete domain.

It should be noted that the longitudinal train dynamics model employed in this study is a linear or quasi-linear approximation, used primarily for state estimation and control optimization within the MPC prediction layer, rather than an exact representation of the train’s dynamics under all operating conditions. The model parameters are obtained through system identification or empirical calibration and can reflect the average dynamic characteristics under typical operating scenarios. However, the model may not fully capture extreme nonlinear behaviors, such as wheel slip, spin, or emergency braking, and the uncertainty in model parameters has not been systematically verified in this study, which may have some impact on control performance. It is worth emphasizing that the inherent rolling optimization and feedback correction mechanism of MPC can partially mitigate the deviations caused by model simplifications and parameter uncertainties, thereby maintaining control performance stability and robustness while ensuring real-time computability.

### 2.3. Constraints and Evaluation Model for Urban Rail Train Tracking Control

In the process of train operation modeling and control design, in addition to accurately describing the train’s dynamic equations, it is also necessary to introduce a series of reasonable assumptions to ensure both the solvability of the model and the feasibility of simulation. These assumptions not only reflect the safety regulations and physical constraints inherent in practical operation but also incorporate appropriate idealizations and simplifications. In this way, the validity of the research outcomes is preserved while avoiding excessive model complexity that would hinder implementation. Specifically, this study clarifies the assumptions made with respect to speed constraints, resistance characteristics, acceleration and deceleration limits, operating mode transitions, and boundary conditions.

**Assumption** **2.** *The train speed at any position must not exceed the line speed limit to prevent safety risks caused by overspeeding. The total resistance acting on the train consists of basic resistance and additional line resistance, providing a more realistic representation of the forces encountered along the track. In addition, the absolute values of acceleration and deceleration must not exceed the prescribed maximum to ensure operational stability and comfort.*

**Assumption** **3.** *The traction and coasting modes cannot be switched directly and must go through an intermediate transition process to avoid sudden impacts on the traction system and passengers. Furthermore, the train’s initial and terminal speeds are both set to zero, the initial time is zero, and the errors in stopping position and time must remain within the allowable limits to ensure that the train can safely stop at the designated location and satisfy scheduling requirements.*

In this study, based on Assumptions 2 and 3, the train operation process can be mathematically described while ensuring both the solvability of the model and the feasibility of simulation. These assumptions provide explicit constraints on speed, resistance, acceleration and deceleration, operating mode transitions, and boundary conditions. Furthermore, the constraints listed in Equation (4) are treated as hard constraints, which must be strictly enforced in train tracking control to ensure operational safety, comfort, punctuality and all.

(4) $$\left\{\begin{array}{c} V\left(x\right)\leq V_{\lim}\left(x\right)\\ W(V,x)=R_{0}\left(V\right)+r_{e}\left(x\right)\\ \left|a(k)\right|\leq {\left|a\right|}_{\max}\\ lr\left(k\right)\leq lr_{\max}\\ oc(k)\times oc(k+1)\neq -1\\ V(0)=V(X)=t(0)=0\\ \left|\bar{D}-X\right|<\Delta S_{\max}\\ \left|\bar{T}-t\left(X\right)\right|<\Delta T_{\max} \end{array}\right.$$

where V(x) and $V_{\lim}\left(x\right)$ represent the actual speed and speed limit at the location point *x*, respectively; $R_{0}\left(V\right)$ and $r_{e}\left(x\right)$ represent the basic resistance under the speed value *V* and the additional resistance of the line at the location point *x*, which includes grade, curve and tunnel resistance, respectively; $\left|a(k)\right|$ denotes the absolute magnitude of the train’s acceleration or deceleration during the *k*-th time cycle, while ${\left|a\right|}_{\max}$ denotes the permissible upper bound for these dynamic responses. Ir(k) represents the rate of impact experienced by the train during the *k*-th cycle; $Ir_{\max}$ represents the maximum train impact rate allowed; oc(k) represents the manoeuvring mode under the *k*-th cycle, and there are five kinds of manoeuvring modes, namely, maximum driving force, partial driving force at constant speed, idling, partial braking force at constant speed, and maximum braking, which are denoted by 1, tc, 0, tb, and −1, respectively, where tc∈(0,1), tb∈(−1,0); *X* and $\bar{D}$ represent the actual and expected running distances of the automatic train operation, respectively; t(X) and $\bar{T}$ represent the actual and expected running times, respectively; $\Delta S_{\max}$ and $\Delta T_{\max}$ correspond to the maximum allowable position error and time error for stopping, respectively.

In addition to the hard constraints described in Equation (4), the urban rail systems must also satisfy supplementary performance constraints to ensure system stability, passenger comfort, and control accuracy. In particular, the integral of time-weighted absolute error (ITAE) is introduced as a commonly used performance index in tracking control. The ITAE provides a quantitative assessment of the tracking performance of automatic train operation. The specific formula are as follows:

(5) $$ITAE=\int_{0}^{\bar{T}}t\left|\delta (t)\right|dt$$

where $\left|\delta (t)\right|$ represents the absolute error between the actual tracking control speed of the train and the target speed under the actual running time *t*.

**Assumption** **4.** *At any position, the error between the controlled speed and the target speed must not exceed the prescribed maximum allowable value to ensure accurate tracking of the target speed profile. Both the control input and its increment should remain within the specified upper and lower bounds to prevent excessive or abrupt controller outputs that could compromise system stability or comfort. In addition, the integral of the absolute value of the ITAE must not exceed the prescribed threshold, ensuring that the cumulative effect of speed tracking errors over the entire operational cycle remains controllable.*

(6) $$\left\{\begin{array}{c} \left|\delta (t)\right|\leq {\left|\delta (t)\right|}_{\max}\\ u_{\min}\leq u\left(k\right)\leq u_{\max}\\ \Delta u_{\min}\leq \Delta u\left(k\right)\leq \Delta u_{\max}\\ ITAE\leq ITAE_{\max} \end{array}\right.$$

where ${\left|\delta (t)\right|}_{\max}$ represents the maximum absolute error in the train’s actual speed relative to the target speed; u(k) and $\Delta u\left(k\right)$ represent the control input and the control increment at the *k*-th time, respectively, here, *k* represents the discrete time index of the control system; $u_{\min}$, $u_{\max}$, $\Delta u_{\min}$ and $\Delta u_{\max}$ represent the upper and lower bounds of the control input and its incremental change, respectively. $ITAE_{\max}$ represents the maximum allowed time times error absolute value.

The urban rail train tracking control inherently requires the simultaneous assessment of multiple performance criteria. Accordingly, this study constructs a multi-objective optimization model that incorporates ITAE, stopping accuracy, punctuality, comfort, and energy consumption as the key control performance indices. The selection of these five objectives is not arbitrary but follows the regulations set by metro operation authorities and is closely related to the constraints defined in Equation (4). Specifically, the ITAE, as a typical performance indicator of trajectory tracking, quantitatively measures the effectiveness of speed curve tracking. The stopping accuracy corresponds to the deviation between the actual and desired stopping positions, ensuring accurate station stops. The punctuality corresponds to the time constraint, ensuring punctual operation. The comfort index corresponds to the acceleration constraint, reflecting the passenger riding experience. The total energy consumption corresponds to the resistance and additional energy demand of the train, reflecting the energy efficiency characteristic imposed by the resistance constraint. By optimizing these five objectives, the proposed model can comprehensively enhance train operation quality, comfort, punctuality, and energy consumption under strict constraint satisfaction. The evaluation model is formulated as follows:

(7) $$\begin{array}{c} \min(ITAE,\Delta S,\Delta t,K_{Jerk},E)\\ \left\{\begin{array}{c} ITAE=\int_{0}^{\bar{T}}t\left|\delta (t)\right|dt\\ \Delta S=\left|\bar{D}-X\right|<\Delta S_{\max}\\ \Delta t=\left|\bar{T}-t\left(X\right)\right|<\Delta t_{\max}\\ K_{Jerk}=\int \left|\Delta a\right|d_{S/\bar{D}}\\ E=\int \left(F_{t}-F_{r}\right)d_{S} \end{array}\right. \end{array}$$

where ΔS is the distance error of the train stopping position point; Δt is the time error of the train stopping position point; $K_{Jerk}$ is adopted as the comfort evaluation index in this study. Its definition is based on prior research and the international standard ISO 2631 [31], which evaluates passenger comfort according to the degree of discomfort induced by vibrations. According to ISO 2631 and related literature, passengers’ perception of comfort is not only influenced by the magnitude of acceleration but is also more sensitive to changes in acceleration. Therefore, in this work, $K_{Jerk}$ is quantitatively defined as the absolute value of acceleration variation per unit distance or per unit time, providing a physically meaningful and widely recognized metric for assessing train comfort. *E* represents the total energy consumption, accounting for both traction and braking forces along the displacement of the train; $d_{S}$ represents a small incremental distance along the train’s path and serves as the integration variable.

Generally, a reduction in ITAE indicates an improvement in the dynamic tracking performance of the train speed profile, which typically contributes to lower energy consumption, enhanced comfort, and improved stopping accuracy and punctuality. However, these performance indices are not fully consistent under different operating conditions and may still involve certain trade-offs; therefore, multiple performance metrics need to be jointly considered in the optimization modeling process.

The ITAE, distance error ΔS, time error Δt, comfort level $K_{Jerk}$, and energy consumption *E* are all evaluation indexes for tracking control of urban rail train, and they need to be made as small as possible [32].

## 3. Multi-Objective Whale Optimization Algorithm

### 3.1. Whale Optimization Algorithm

The whale optimization algorithm (WOA) is a nature-inspired optimization technique that simulates the foraging behavior of humpback whales and exhibits excellent optimization performance with strong global search capabilities [33]. During hunting, humpback whales not only encircle their prey but also approach it along a spiral-shaped trajectory while simultaneously shrinking the encircling circle.

The core idea of the basic WOA can be summarized into three main behavioral mechanisms:

(I) Encircling prey behavior: whales determine the distance to their prey from their current location and subsequently adjust their path to move toward it. This process can be mathematically represented as follows:

(8) $$x\left(t+1\right)=\left\{\begin{array}{cc} x^{*}\left(t\right)-H\cdot D & p<P_{s}\\ x^{*}\left(t\right)+D_{p}\cdot e^{bt}\cdot \cos\left(2\pi l\right) & p\geq P_{s} \end{array}\right.$$

where $D_{P}=\left|x^{*}\left(t\right)-x\left(t\right)\right|$ represents the distance between the whale’s position x(t) and the best solution $x^{*}\left(t\right)$; $x^{*}\left(t\right)$ represents the position vector of the current optimal solution; $D=\left|Cx_{rand}-x\left(t\right)\right|$ represents the absolute value of the difference between $Cx_{rand}$ and whale x(t), $Cx_{rand}$ represents the position vector of a randomly selected whale, *H* and *C* represent correlation coefficients, $H=2a\times r_{1}-a$, $C=2\times r_{2}$, $r_{1}$ and $r_{2}$ represent random numbers within the interval (0, 1); *a* represents the convergence factor. *p* represents the behavior selection probability of the whale, $p\in \left[0,1\right]$; $P_{s}$ represents the probability that the whale chooses to attempt encircling prey behavior, $P_{s}\in \left[0,1\right]$, $1-P_{s}$ represents the probability that the whale chooses to perform bubble-net spiraling hunting behavior; *b* represents the coefficient that controls the spiral shape adjustment, generally a constant 1; *l* represents a randomly chosen value within the interval (−1, 1).

(II) Prey search behavior: During the hunting process, humpback whales randomly search for prey. In the basic WOA, when H≥1, A leading individual in the search process $x_{rand}$ is chosen randomly and the other whales update their positions based on the leader’s location. This guides the whales away from the current prey in order to find more suitable targets, aiming to enhance the algorithm’s global search ability. The position update formula for whale individuals performing random prey search in the basic WOA is presented below:

(9) $$x\left(t+1\right)=x_{rand}-H\cdot D$$

(III) Spiral position update: this simulates the whale approaching the prey along a spiral path, enhancing local search capabilities.

In summary, the WOA constructs an optimization framework that balances exploration and exploitation through the coordinated operation of the three core mechanisms described above. However, during the execution of the basic WOA, a linearly decreasing convergence factor is typically introduced to further enhance the algorithm’s adaptability and robustness.

(10) $$a=2-2\times t/T_{\max}$$

where *t* is the present optimization iteration count, $T_{\max}$ is the maximum optimization iteration count.

### 3.2. Tchebycheff Decomposition for Multi-Objective Optimization

Through decomposition, the inherently complex nature of multi-objective problems are reduced by breaking them down into scalarized subproblems that are easier to solve. Among these, the Tchebycheff decomposition method is particularly effective. The specific formulation of the sub-optimization problem based on the Tchebycheff decomposition is described as follows:

(11) $$\min\;\;\;\;g^{te}\;\left(x|\lambda ,Z^{*}\right)=\max_{1\leq i\leq n}\left\{\lambda_{i}\left|f_{i}\left(x\right)-Z_{i}^{*}\right|\right\}s.t.\;\;\;\;x\in \Omega$$

where $Z^{*}={\left(Z_{1}^{*},Z_{2}^{*},\ldots ,Z_{n}^{*}\right)}^{T}$ represents the point of reference; $Z_{i}^{*}=\min\left\{f_{i}\left(x\right)|x\in \Omega \right\},i=1,\ldots ,n$ represents the minimum value of each objective function up to the current point is taken as the optimal solution, *n* is the amount of objectives, *x* is the variables that determine the outcome; $f_{i}\left(x\right)=\frac{f_{i}^{\prime }\left(x\right)-A_{i}}{B_{i}-A_{i}}$, $f_{i}\left(x\right)$ and $f_{i}^{\prime }\left(x\right)$ represent the i-th objective after and before normalization, respectively; while $B_{i}$ and $A_{i}$ represent the corresponding tolerance values, specifically the appropriate upper and lower bounds; λ represents the weight vector; $\lambda_{i}$ represents the weight corresponding to the *i*-th objective; $\lambda_{i}>0,\sum_{i=1}^{n}\lambda_{i}=1$; $g^{te}\left(x|\lambda ,Z^{*}\right)$ is the value of the aggregation function for decision variable *x* under the vector of weights λ and reference value $Z^{*}$.

For the single-objective subproblems derived from the Tchebycheff decomposition, the calculation formula for the weight corresponding to each objective in the weight vector is given as follows:

(12) $$\lambda_{i}=\frac{1}{f_{i}\left(x\right)-Z_{i}^{*}}(\sum_{k=1}^{n}\frac{1}{f_{k}\left(x\right)-Z_{k}^{*}}{)}^{-1}$$

The optimal solution $x^{*}$ of the single-objective subproblem derived from the Tchebycheff decomposition with respect to the weight vector λ corresponds to the Pareto optimal solution. It represents the unique intersection point between the line $\frac{f_{1}-Z_{1}^{*}}{\lambda_{1}}=\frac{f_{2}-Z_{2}^{*}}{\lambda_{2}}=\ldots =\frac{f_{n}-Z_{n}^{*}}{\lambda_{n}}$ in the *n*-dimensional space and the pareto front.

The decomposition-based method yields a group of mutually non-dominated optimal solutions corresponding to different weight vectors, making it difficult to select a preferred solution. To address this, a predefined objective weight vector $\lambda_{T}$ is required, which is composed of the weight coefficients $\omega_{i}$ assigned to each optimization objective $g_{i}$. $\lambda_{T}={\left(\omega_{1},\omega_{2},\ldots ,\omega_{n}\right)}^{T}$, the corresponding weight coefficient $\omega_{i}$ reflects the relative importance of the optimization objective $f_{i}$. Typically, $\sum_{i=1}^{n}\omega_{i}=1$ is set. The final optimal solution *x* is selected from the set of optimized solutions $\Omega_{0}$ as the one whose weight vector $\lambda_{x}$ is closest to the objective weight vector $\lambda_{T}$, that is, the following equation holds.

(13) $$\cos\langle \lambda_{x},\lambda_{T}\rangle =\max_{y\in \Omega_{E}}\left(\cos<\lambda_{y},\lambda_{T}>\right)$$

where $\cos<\lambda_{x},\lambda_{T}>$ represents the cosine of the angle between the weight vector $\lambda_{x}$ of the optimal solution *x* and the target weight vector $\lambda_{T}$.

### 3.3. Improved Multi-Objective Whale Optimization Algorithm

Building upon the fundamental mechanisms of the traditional WOA, this paper introduces a multi-objective whale optimization algorithm (MOWOA). This algorithm introduces a nonlinearly decreasing convergence factor based on the natural constant, as well as a variable rate adjustment factor. Additionally, to address complex multi-objective optimization problems, the Tchebycheff decomposition method is employed to decompose them into multiple independent single-objective subproblems, Compared with the weighted sum method and the boundary intersection approach, Tchebycheff decomposition is capable of effectively handling non-convex Pareto fronts and is insensitive to differences in the scales and dimensions of objective functions, thereby exhibiting favorable numerical stability. In addition, this method can be readily integrated with the rolling optimization mechanism of MPC, enabling improvements in trajectory tracking accuracy and control performance while maintaining optimization effectiveness and engineering feasibility. By employing this decomposition approach, MOWOA enhances its ability for both global search and local refinement, thereby improving the algorithm’s performance in multi-objective optimization. The specific formula for calculating the convergence factor *a* is given as follows:

(14) $$a\left(t\right)=-0.5+2.5\times e^{{\left(\frac{t}{T_{\max}}\right)}^{\beta a}\cdot \ln\left(\frac{0.5}{2.5}\right)}$$

where $\beta_{a}$ is the variable rate adjustment factor used for the nonlinear decrease of the convergence factor *a*. The adaptive decreasing trend of *a* can be adjusted through the factor $\beta_{a}$. It is important to select a practical and problem-specific value of $\beta_{a}$ based on the characteristics of the optimization problem, to achieve better optimization performance. Therefore, this variable rate adjustment strategy can dynamically balance the convergence rate and convergence accuracy of the proposed method during the iteration process, thereby enhancing its global convergence capability to the greatest extent possible.

To enhance the tracking performance of the MPC under complex and dynamic operating conditions in urban rail systems, this paper proposes a method using MOWOA for optimizing control parameters. The design highlights of MOWOA lie in the introduction of a nonlinearly decreasing convergence factor based on the natural constant and a variable rate adjustment mechanism. Meanwhile, the Tchebycheff decomposition method is employed to transform the multi-objective optimization problem into multiple independent single-objective subproblems. These enhancements improve the global search and local refinement capabilities of the optimization process, allowing the MPC to obtain better-initialized parameters.

**Figure 2:**
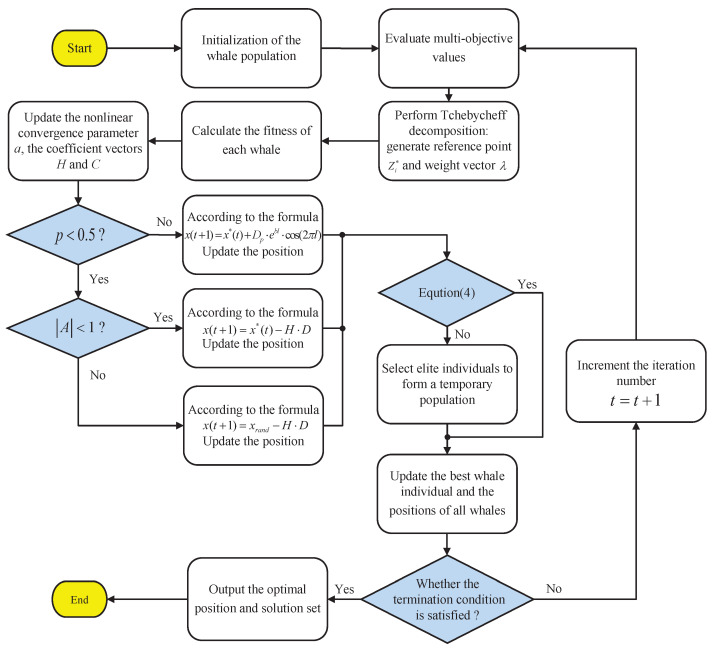
The flowchart of the MOWOA proposed in this study.

As shown in Figure 2, the overall optimization procedure of the MOWOA can be summarized as follows. The algorithm initializes the whale population and evaluates the multi-objective function values of each individual. The Tchebycheff decomposition method is then applied to construct reference points and weight vectors, converting the multi-objective problem into a set of scalar subproblems for fitness evaluation. During the iterative process, a nonlinearly decreasing convergence parameter based on the natural constant and dynamically updated coefficient vectors are used to regulate the search behavior, enabling alternation among encircling, random exploration, and spiral updating strategies to balance global exploration and local exploitation. An elite selection mechanism is further introduced to update the best individual and the population positions. Upon meeting the termination condition, the algorithm outputs the optimal solution set, thereby providing well-initialized MPC parameters and enhancing the tracking control performance of urban rail trains under complex operating conditions.

### 3.4. The Tuning Framework of Control Parameters for Multi-Objective Performance Optimization

The proposed MPC, which features simultaneous online adjustment of the control parameter softening factor and correction coefficient, encompasses seven parameters to be tuned. These parameters include the maximum value of the softening factor $a_{\max}$, the minimum value of the softening factor $a_{\min}$, the trend control factor of the softening factor bα, the speed error quantization factor $k_{in\_e}$, the initial value of the correction coefficient $\alpha h_{i}$, the speed error rate quantization factor $k_{in\_\dot{e}}$, and the proportional adjustment factor of the correction coefficient $k_{out\_\Delta \alpha h}$, along with the shape of their membership functions. The goal of tuning these parameters is to simultaneously optimize key evaluation indicators, including the distance error (Δs), time error (Δt), comfort index ($K_{Jerk}$), traction energy consumption (*E*), and the ITAE, to ensure optimal train tracking performance under varying operating conditions.

To achieve optimal automatic train tracking performance, the MOWOA based on Tchebycheff decomposition is employed to optimize and tune an initial set of control parameters. During the optimization process, whenever a candidate set of initial parameters requires evaluation, the optimization is temporarily paused. The urban rail train tracking control algorithm is then executed using this parameter set until the corresponding performance indicators are obtained.

**Figure 3:**
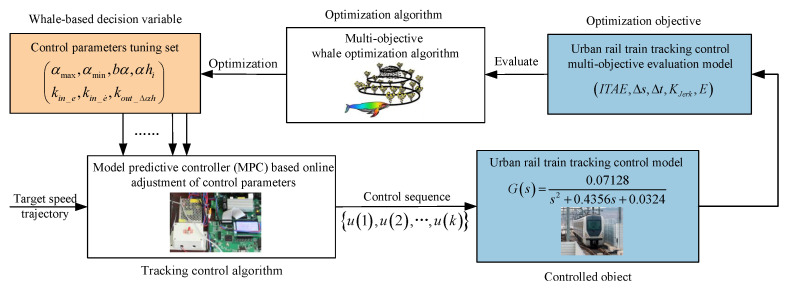
Schematic diagram of the MPC-based urban rail train tracking control system using MOWOA for control parameter optimization.

In essence, MOWOA iteratively generates optimized solutions, while the tracking control algorithm evaluates each solution by simulating the tracking process under real urban rail operating scenarios using predefined target speed trajectory. The quality of each solution is assessed based on the Pareto optimality principle, the Tchebycheff aggregation function value, and its cosine similarity with the target weight vector ($\lambda_{T}$). The MOWOA is primarily employed for the offline self-tuning of key MPC parameters, while the MPC performs rolling optimization and real-time control during online operation based on these optimized parameters, thereby balancing control performance with computational efficiency. The schematic diagram illustrating the tuning process of initial control parameters based on the MOWOA is shown in Figure 3.

## 4. Model Predictive Controller

### 4.1. Dynamic Matrix Prediction Model

In recent years, the MPC has emerged as a powerful control strategy in urban rail transit systems due to its ability to predict future behavior and optimize control actions within system constraints. For the train tracking control, where precise speed and position tracking are essential for safety and energy efficiency, the MPC provides a systematic framework to handle multi-variable dynamics and time-varying operating conditions.

Among the many variants of MPC, dynamic matrix control (DMC) is one of the earliest and most practically implemented algorithms [34]. Although the proposed control strategy in this study is referred to as a model predictive controller, the implementation specifically adopts the DMC approach. This paper proposes MPC-MOWOA that incorporates online adjustment of control parameters to enhance adaptability and tracking performance in dynamic urban rail environments.

The DMC uses the step response of the system as the internal model to predict future outputs, which enables accurate modeling of plant dynamics using only a finite number of response samples. Given a modeling horizon of length *N*, the system’s dynamic properties can be described using the *N* discrete samples $\left\{a_{1},a_{2},\ldots ,a_{N}\right\}$ obtained from its unit step response. The prediction of the model’s output is carried out via the following formulation.

(15) $$Y_{P}\left(k\right)=Y_{0}\left(k\right)+A\Delta R\left(k-1\right)$$

where $Y_{p}\left(k\right)=\left[\begin{array}{c} y_{p}\left(k+1|k\right),\\ y_{p}\left(k+2|k\right),\\ \ldots ,\\ y_{p}\left(k+N|k\right) \end{array}\right]$ represents the predicted output generated by the model at time step *k*; $Y_{0}\left(k\right)=\left[\begin{array}{c} y_{0}\left(k+1|k\right),\\ y_{0}\left(k+2|k\right),\\ \ldots ,\\ y_{0}\left(k+N|k\right) \end{array}\right]$ represents the initial value of the model; $\Delta U\left(k-1\right)=\left[\begin{array}{c} \Delta u(k)\\ \Delta u(k+1)\\ \ldots ,\\ \Delta u(k+N-1) \end{array}\right]$ represents the control incremental sequence vector; $A=\left[\begin{array}{cccc} a_{1} & 0 & L & 0\\ a_{2} & a_{1} & L & 0\\ M & M & 0 & M\\ a_{N} & a_{N-1} & L & a_{1} \end{array}\right]$ represents the dynamic matrix, *i* represents the index value at some future time in the modeling time domain, $i\in \left\{1,2,\ldots ,N\right\}$.

### 4.2. Rolling Optimization

To effectively avoid severe fluctuations during the control process, the current output value y(k) of the DMC system should follow a predefined smooth reference trajectory toward the target value $y_{r}$, thereby enhancing the robustness of the system. A commonly used form of such a reference trajectory is described below.

(16) $$y_{f}\left(k+i\right)=a^{i}y\left(k\right)+\left(1-a^{i}\right)y_{r}$$

where $y_{f}\left(k+i\right)$ represents the final output value expected to be achieved; $a^{i}$ represents the *i*-th softening factor, $a^{i}\in \left[0,1\right)$; y(k) represents the actual output value of the system at the current control cycle (the *k*-th cycle); and $y_{r}$ represents the reference target value of the system, which is also the setpoint input value [35].

The formulation of a secondary objective function is critical to enhancing tracking performance within the rolling optimization framework of DMC. Assuming the length of the optimization horizon is *P*, the length of the control horizon is *L*, and L≤P≤N, the following presents the detailed formulation of the secondary objective.

(17) $$\begin{array}{c} J={∥Y_{p}\left(k\right)-Y_{f}\left(k\right)∥}_{Q}^{2}+{∥\Delta U_{L}\left(k-1\right)∥}_{R}^{2}\\ =\sum_{i=1}^{P}q_{i}\left[y_{p}\left(k+i\right)\left|k\right)-y_{f}\left(k+i\right)\right]+\sum_{i=1}^{L}r_{i}\Delta u\left(k+i-1\right) \end{array}$$

where $Q=diag{\left[q_{1},q_{2},\ldots ,q_{p}\right]}^{T}$ denotes the error weighting matrix; $R=diag{\left[r_{1},r_{2},\ldots ,r_{L}\right]}^{T}$ denotes the control increment weighting coefficient matrix; $Y_{f}\left(k\right)=[y_{f}\left(k+1\right),y_{f}\left(k+2\right),\ldots ,$$y_{f}\left(k+P\right)]$ denotes the anticipated control inputs sequence.

According to the extreme value theory, under unconstrained conditions, the essential condition for the objective function *Y* to achieve its minimum is $\frac{\partial J}{\partial \Delta U_{L}\left(k\right)}=0$. Thus, the optimal solution for the control sequence can be obtained through receding horizon optimization [36]. The optimal control sequence is computed according to the following formula.

(18) $$\Delta U_{L}\left(k-1\right)={\left(A^{T}QA+R\right)}^{-1}A^{T}Q\left[Y_{f}\left(k\right)-Y_{0}\left(k\right)\right]$$

On this basis, the actual control input u(k) is obtained. The detailed computational equation for the u(k) is described as follows.

(19) u(k)=u(k−1)+Δu(k−1)

During the following control interval, the k+1-th cycle, the matching Δu(k) and Δu(k+1) is obtained using the aforementioned method. By repeating this process iteratively, rolling optimization of the actual control inputs can be achieved.

Although the current design does not explicitly include Lyapunov-based terminal constraints, these could be incorporated in future extensions to ensure closed-loop stability while optimizing multi-objective performance.

### 4.3. Feedback Correction

Any practical control system is subject to stochastic and uncertain disturbances, which increase the difference between the system’s real-time output and the expected reference, potentially leading to system instability. Feedback correction is a critical component of predictive control and is employed to mitigate the impact of disturbances and uncertainties on control performance, thereby achieving the desired control objectives.

The error δ(k), representing the discrepancy between the real-time output y(k) and its predicted counterpart $y_{p}\left(k\right)$ at step *k*, is computed as.

(20) $$\delta \left(k\right)=y\left(k\right)-y_{p}\left(k\right)$$

Incorporating feedback correction, the predicted output $y_{p2}\left(k\right)$ is accordingly modified. The corresponding correction formula is formulated as follows:

(21) $$y_{p2}\left(k\right)=y_{p}\left(k\right)+H\delta \left(k\right)=y_{N0}\left(k\right)+A\Delta U\left(k-1\right)+H\delta \left(k\right)$$

where *H* represents the correction vector.

If the filter is designed in a first-order form, by $G_{F}(z)=\frac{\alpha h}{1+\left(\alpha h-1\right)z^{-1}}\frac{\partial^{2}\Omega }{\partial u^{2}}$, the correction coefficients are represented αh. In the context of the automatic train operation (ATO) system, the filter in the feedback correction part is typically designed as a first-order filter. In this case, the correction vector *H* is determined using the method $\left[1,\alpha h,\ldots ,\alpha h\right]$. Here, the length of the correction vector *H* is *N*, and αh∈(0,1).

In the *k*-th cycle, the future prediction time points are shifted to [k+1,…,k+N]. This means that the prediction values for the *k*-th cycle can be obtained by simply shifting $y_{p2}\left(k\right)$, $y_{N0}\left(k\right)=S\cdot y_{p2}\left(k\right)$. Here, *S* represents the transformation matrix, where $S=\left[\begin{array}{cccc} 0 & 1 & L & 0\\ M & 0 & L & 0\\ M & M & 0 & 1\\ 0 & L & L & 1 \end{array}\right]$.

### 4.4. Online Adjustment of Softening Factor

In practical engineering applications, the urban rail train tracking control problem is inherently fuzzy in nature. To address this issue, a fuzzy satisfaction function is defined to quantify the degree to which the control objectives are achieved, and the control problem is reformulated as an optimization-based decision-making problem through fuzzy inference [37]. Specifically, the fuzzy satisfaction function consists of two components.

The first component is the system response time, which reflects the dynamic tracking capability of the control system with respect to the reference command. Its magnitude is closely related to the error between the actual output and the setpoint, as well as the rate of change of this error. The second component is the fuzzy satisfaction of the predicted outputs. At each sampling instant, the outputs at future time instants within the prediction horizon are evaluated, and corresponding membership functions are defined to characterize the degree of closeness between the predicted system behavior and the desired trajectory.

By combining these two components, a comprehensive fuzzy satisfaction index is obtained, which serves as a quantitative measure of the overall control performance. Based on this index, the softening factor can be adjusted online in real time, enabling the control system to adaptively balance speed tracking accuracy, energy consumption optimization, and ride comfort during dynamic operation, thereby enhancing the flexibility and stability of trajectory tracking control.

First, The system response time is related to the error between the actual output and the setpoint, as well as the rate of change of the error. For any given sampling time *t*, the estimated system response time $t_{s}\left(t\right)$ is calculated using the following formula:

(22) $$t_{s}\left(t\right)=\left\{\begin{array}{ll} 0 & \dot{\delta }\left(t\right)=0,\delta \left(t\right)=0\\ \frac{\delta \left(t\right)}{\left|\dot{\delta }\left(t\right)\right|} & \dot{\delta }\left(t\right)<0\\ M & \dot{\delta }\left(t\right)\geq 0,\delta \left(t\right)\neq 0 \end{array}\right.$$

where δ(t) and $\dot{\delta }\left(t\right)$ denote the error between the actual output and the setpoint, as well as the variation rate of the error signal, respectively; $t_{s}\left(t\right)$ denotes the estimated system response time, defined as the time required for the system to reach the setpoint based on the absolute value of the current error derivative $|\dot{\delta }\left(t\right)|$.

When conditions $\delta \left(t\right)=o,\dot{\delta }\left(t\right)=0$ are satisfied, the system is considered to be in a stable state, with a system response time of zero. When condition $\dot{\delta }\left(t\right)<0$ occurs, the deviation from the setpoint in the system output shows a decreasing trend, and the system response time is calculated using formula $\frac{\delta \left(t\right)}{\left|\dot{\delta }\left(t\right)\right|}$. When conditions $\dot{\delta }\left(t\right)\geq 0,\delta \left(t\right)\neq 0$ are fulfilled, the deviation from the setpoint in the system output shows an increasing trend, indicating that the system output requires a relatively long time to reach the setpoint. Therefore, the system response time is assigned a large positive value *M*.

**Figure 4:**
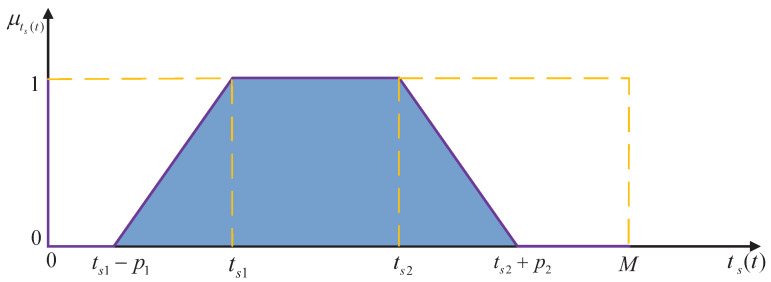
Diagram for membership function of estimated system response $t_{s}\left(t\right)$ about fuzzy satisfaction degree.

Based on the inferred system response duration *t* at time $t_{s}\left(t\right)$, a fuzzy mapping function can be constructed to evaluate the degree of satisfaction with respect to how well the response time aligns with control objectives.

(23) $$\mu_{t_{s}\left(t\right)}=\left\{\begin{array}{ll} 1, & t_{s}\left(t\right)=0\\ 0, & t_{s}\left(t\right)<t_{s1}-p_{1}\\ 1+\frac{t_{s}\left(t\right)-t_{s1}}{p_{1}}, & t_{s1}-p_{1}\leq t_{s}\left(t\right)<t_{s2}\\ 1, & t_{s1}\leq t_{s}\left(t\right)<t_{s2}\\ 1-\frac{t_{s}\left(t\right)-t_{s2}}{p_{2}}, & t_{s2}\leq t_{s}\left(t\right)<t_{s2}+p_{2}\\ 0, & t_{s2}+p_{2}\leq t_{s}\left(t\right) \end{array}\right.$$

where $p_{1}$ and $p_{2}$ represent the fuzzy widths, indicating the degree to which the design requirements are satisfied. $t_{s1}$ and $t_{s2}$ represent the upper and lower bounds, respectively, of the desired system response time. As depicted in Figure 4, the fuzzy satisfaction level $\mu_{t_{s}\left(t\right)}$ is defined over the estimated system response time $t_{s}\left(t\right)$ via a membership function.

**Figure 5:**
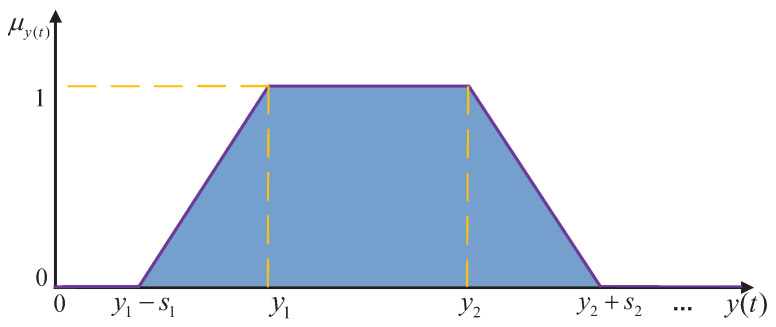
Diagram for membership function of predictive output y(t) about fuzzy satisfaction degree.

Second, a corresponding satisfaction function can be formulated for the forecasted outputs y(t+i) across future prediction steps following time *t*.

(24) $$\mu_{y(t)}=\left\{\begin{array}{ll} 0, & y\left(t\right)<y_{1}-s_{1}\\ 1+\frac{y\left(t\right)-y_{1}}{s_{1}}, & y_{1}-s_{1}\leq y\left(t\right)<y_{1}\\ 1, & y_{1}\leq y\left(t\right)\leq y_{2}\\ 1-\frac{y\left(t\right)-y_{2}}{s_{2}}, & y_{2}\leq y\left(t\right)\leq y_{2}+s_{2}\\ 0, & y_{2}+s_{2}\leq y\left(t\right) \end{array}\right.$$

where $t_{1}$ and $t_{2}$ represent the fuzzy widths, indicating the degree to which the system meets the designer’s requirements. If $s_{1}$ and $s_{2}$ are both 0, it implies that the requirements for the system control objectives are strict. In the context of ATO tracking control, neither $s_{1}$ nor $s_{2}$ would be zero. $y_{1}$ and $y_{2}$ represent the upper and lower bounds of the desired design values, respectively. The membership function diagram illustrating the relationship between the forecasted output value y(t) and its fuzzy satisfaction degree $\mu_{y(t)}$ is shown in Figure 5.

Based on fuzzy satisfaction degrees $\mu_{y(t+1)},\ldots ,\mu_{y(t+p)}$, and fuzzy inference can be applied to obtain the fuzzy satisfaction index $\mu_{\min}$ for the control system’s performance in meeting the control objectives at the sampling time.

(25) $$\mu_{\min}=\mu_{t_{s}\left(t\right)}\wedge \min\left\{\mu_{y(t+1)},\mu_{y(t+2)},\ldots ,\mu_{y(t+p)}\right\}$$

If the fuzzy satisfaction index $\mu_{\min}$ is larger, it indicates that the system error is smaller, but the response time is longer. In this case, the softening factor should be appropriately reduced. On the contrary, if the fuzzy satisfaction index $\mu_{y(k)}$ is smaller, when the system exhibits a larger control error despite a shorter response time, it becomes necessary to moderately increase the softening factor. To this end, an online adjustment mechanism for $\alpha (\mu_{\min})$ is developed based on the fuzzy satisfaction index $\mu_{\min}$. The corresponding computational expression is formulated as follows.

(26) $$\alpha \left(\mu_{\min}\right)=\alpha_{\max}\cdot e^{{\mu_{\min}}^{b\alpha }\ln\left(\frac{\alpha_{\min}}{\alpha_{\max}}\right)}$$

where $\alpha_{\max}$ and $\alpha_{\min}$ denote the upper and lower limits of the softening coefficient, respectively. The term $\ln(\frac{\alpha_{\min}}{\alpha_{\max}})$ acts as the gain parameter, influenced jointly by these boundary values. The variable bα serves as a shaping factor that governs the curvature of the adjustment function. *e* is the natural logarithm, where e=2.71828.

### 4.5. Online Adjustment of the Correction Coefficient

In general, for the feedback filter $G_{F}\left(z\right)$ the larger the correction coefficient αh, the slower the system response, and the less sensitive it is to model mismatch; conversely, the smaller the correction coefficient αh, the faster the system response, but the more likely it is to cause system oscillations. Based on this, this paper proposes an online adjustment strategy for the correction coefficient. When the absolute error is large and the error is rapidly decreasing, as indicated by a relatively large negative rate of change, the system is responding too quickly. In this case, the response speed should be moderately reduced to avoid inducing oscillations, and the calibration parameter should be minimized. On the other hand, when the error is small but increasing rapidly, which corresponds to a large positive rate of change, it indicates a slow response. To prevent error amplification, the system’s response speed should be appropriately increased, and the calibration parameter should be set to a larger value. The correction coefficient adjustment Δαh is determined jointly by the absolute value of the error δ and the rate of change of the error $\dot{\delta }$, The specific fuzzy control rules are shown in Table 2.

The Table 2 defines the fuzzy inference rules used to determine the adjustment quantity of the correction coefficient based on the error δ and the error variation rate $\dot{\delta }$. Each table cell corresponds to a fuzzy rule output linked to linguistic labels spanning from negative big to positive big. The rules are constructed to ensure that the correction coefficient is adjusted adaptively according to the system’s error dynamics, thereby improving control performance.

The calculation formula for the online-adjusted correction coefficient is given as follows:

(27) $$\alpha h=\alpha h_{i}+\Delta \alpha h$$

where $\alpha h_{i}$ represents the initial value of the correction coefficient.

**Figure 6:**
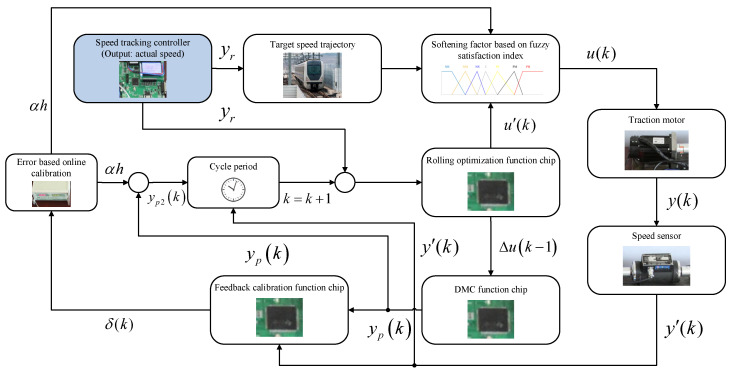
The DMC principle diagram in this study.

As illustrated in Figure 6, the block diagram of the DMC controller is presented to provide a clear and intuitive understanding of its working principle and the interactions among its constituent modules. The diagram depicts the computational logic of the control process, including future output prediction, error evaluation, and the derivation of the optimal control sequence.

## 5. Hardware-in-the-Loop Simulation Experiment for Urban Rail Train Tracking Control

### 5.1. Primary Parameters for Urban Rail Train Tracking Control

This study adopts the scenario from the Dalian Urban Rail Transit Line 12, specifically the section between Lushun New Port and Tieshan Town, as the simulation object. The Dalian Rail Transit Line 12 extends from Hekou in Dalian High-Tech Industrial Zone to Lushun New Port in the Lvshun Economic and Technological Development Zone, comprising a total of eight stations. The main parameters and idealized operation results for urban rail train tracking control are listed in Table 3 and Table 4, respectively. In this study, the expected running time refers to the time required for a train to travel from the departure of the previous station to the arrival at the next station according to the schedule under normal operating conditions. This time is determined by the urban rail transit operator based on multiple factors, including line design, signaling systems, inter-station distance, and vehicle performance, and is approved by the relevant authorities as the basis for train scheduling and operation. The operations department is responsible for executing train operations according to this scheduled time. In train tracking control, the primary objective is to ensure that the actual arrival time closely follows the expected running time, thereby guaranteeing both operational safety and comfort.

**Figure 7:**
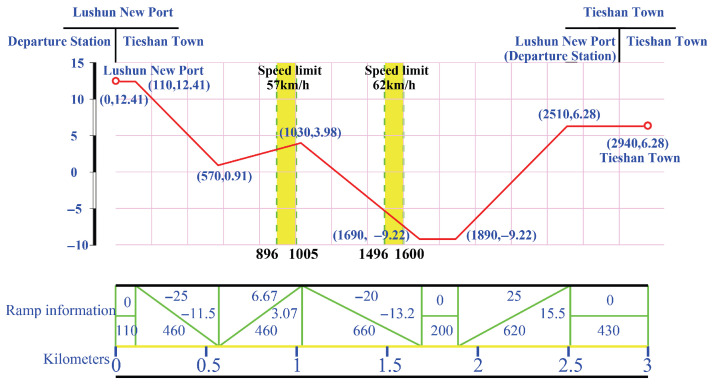
The diagram for ramp path from Lvshun New Port to Tieshan Town.

As illustrated in Figure 7, the longitudinal profile of the section from Lvshun New Port to Tieshan Town comprises multiple gradient segments, speed-restricted zones, and station-related constraints. These factors jointly define the admissible speed envelope during train operation. The figure explicitly presents the spatial distribution of track gradients, the locations of speed limits, and station positions along the route distance, thereby providing the fundamental line characteristics and constraint inputs required for train speed trajectory planning.

**Figure 8:**
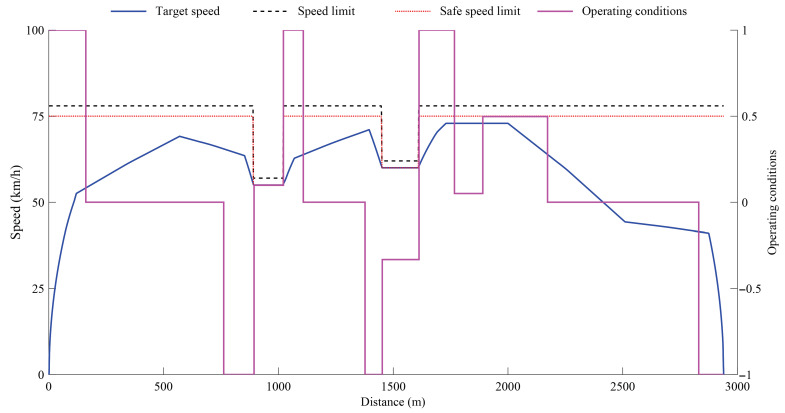
The Limit speed, target speed trajectories and operating condition distance curves of the scenario in this study.

As shown in Figure 8, the planned target speed trajectory strictly complies with both the maximum permissible line speed and the safety speed limits over the entire route. Pronounced deceleration behaviors are observed in speed-restricted sections and station approach areas, reflecting the imposed operational safety and stopping requirements. In contrast, within unconstrained sections, the train speed increases smoothly to balance operational efficiency and passenger comfort. Meanwhile, the corresponding operating-condition-distance curve characterizes the switching process among different operating modes, including traction, cruising, coasting, and braking. Consequently, the target speed trajectory and the operating-condition curve jointly constitute the ideal reference profiles adopted in the subsequent train speed tracking control design.

It should be noted that the above target speed trajectory is obtained under nominal operating conditions and idealized model assumptions. In practical train speed tracking control, the system is inevitably affected by variations in train mass, fluctuations in track resistance, uncertainties in vehicle dynamic parameters, and external disturbances. In addition, model mismatches and measurement errors may persist within the control system, leading to localized speed fluctuations along certain route segments. Therefore, in engineering practice, a reasonable level of deviation between the actual speed trajectory and the target profile is generally acceptable. The essential objective of the control strategy is to effectively suppress error growth and maintain the actual tracking trajectory in close proximity to the reference trajectory, thereby ensuring safe, smooth, and high-performance train operation.

### 5.2. Parameter Settings of the Scenario in This Study

To meet the practical requirements set by locomotive depots and metro operators, the performance parameters listed in Table 5 are determined based on actual operational standards. Specifically, the route length of 2940 m is obtained through field measurements, and the scheduled travel time of 195 s is defined according to the dispatching plan. The five performance indicators, including energy consumption, comfort level, tracking control comfort, distance error, and time error, are derived from routine operational data and validated computational models. These parameters serve as essential references for evaluating system performance and supporting subsequent optimization and control strategy development.

In this study, the parameters of the MPC and the MOWOA were derived from validated empirical values found in relevant literature and then appropriately refined to align with the operational objectives and constraints of the proposed control system [38]. Specifically, the following parameters were used: the train speed sampling period is 0.5 ms, with a control period of 1 ms. However, in practical engineering applications, the control cycle may be extended due to limitations in hardware processing capability and the presence of communication delays. Even so, it can still meet the typical requirements of actual train management systems, with control cycles of 50 ms or 100 ms, thereby ensuring rail vehicle speed tracking accuracy and overall system stability. The model length is set to 60, and both the prediction horizon and control horizon are set to 15. It should be noted that the model length, prediction horizon, and control horizon are all defined in units of discrete sampling steps (dimensionless), where each step corresponds to a sampling period of 0.5 ms. Therefore, the model horizon is equivalent to 60×1ms=60ms, while both the prediction and control horizons correspond to 15×1ms=15ms. The fuzzification width parameters are $p_{1}$ and $p_{2}$ as 0.003 s, and $s_{1}$ and $s_{2}$ as 0.05 km/h. The expected upper and lower bound values for $t_{s1}$ and $t_{s2}$ are 0.005 s and 0.08 s, respectively, and for $y_{\min}$ and $y_{\max}$, they are $y_{r}+0.05$ km/h and $y_{r}-0.05$ km/h. The target speed $y_{r}$ is also defined. The whale population size NPw is 40, the number of iterations $k_{\max}$ is 100, the probability of whales selecting prey-encircling behavior $P_{s}$ is 0.6, and the rate adjustment factor $\beta_{a}$ for the convergence factor *a* is 1.72 [39].

**Figure 9:**
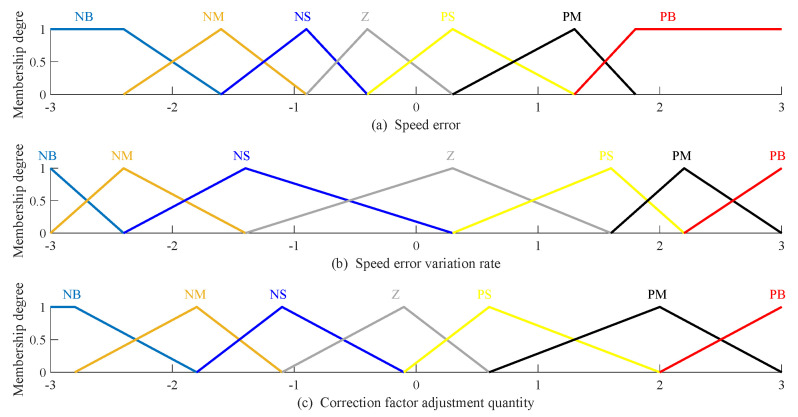
The diagram for variables’ membership functions about correction factor online adjusting.

On this basis, in order to further improve system performance in terms of the five defined indicators, the control parameters of the MPC were optimized using the MOWOA. The initial parameter values were selected based on validated empirical studies and relevant literature, and were then refined through MOWOA to meet the multi-objective performance requirements of the proposed control system. The optimized control parameters are shown in Table 6. These parameters are fine-tuned to adaptively meet the multi-objective performance requirements under varying urban rail operating conditions. Furthermore, the corresponding membership functions for speed error, speed error rate, and correction coefficient adjustments are illustrated in Figure 9 below.

### 5.3. Design and Configuration of the Hardware-in-the-Loop Simulation Experiment

In this study, a hardware-in-the-loop simulation experiment (HILSE) incorporating a traction motor was established and implemented to validate the effectiveness of the proposed MPC strategy for urban rail train operation. The HILSE essentially constructs a virtual train operating environment, in which motor speed tracking is realized under a predefined speed scaling relationship, thereby emulating the train speed trajectory tracking process. Compared with purely numerical simulations conducted in a Simulink environment, the HILSE approach transforms the idealized signal connections and functional blocks in the control model into a physical closed-loop system consisting of real measurement, computation, actuation, and mechanical load components. As a result, a complete practical signal transmission chain and energy flow path are introduced into the control process, enabling the experimental platform to more closely resemble real-world engineering systems.

In practical urban rail applications, train speed trajectory tracking involves multiple performance objectives, time delays, and nonlinearities. Disturbances arise from both external operating conditions and intrinsic system characteristics. By integrating physical hardware into the simulation, the HIL methodology enhances the realism and credibility of controller performance evaluation compared to model-based simulations alone. The experimental platform, incorporating a host computer and HMI, enables control parameter deployment, data acquisition, and real-time monitoring, while speed commands, mode switching, and emergency stop interlocks ensure operability, repeatability, and safety-features often idealized in pure simulations. Moreover, the PMSM speed regulation system exhibits multi-objective dynamics, inherent delays, and strong nonlinearities, including electromagnetic effects (e.g., magnetic saturation) and mechanical influences (e.g., bearing friction, structural vibration), which are the main sources of disturbances and introduce uncertainties into the HILSE for urban rail train speed tracking.

The HILSE involve physical components such as controllers, and typically require embedding the control algorithm into the controller’s core chip while constructing a corresponding virtual trains environments [40]. This setup enables a more effective performance evaluation of the control strategy. The HILSE for urban rail train tracking control offers several notable advantages. First, it can realistically simulate network transmission delays as well as packet errors or losses. Second, it is capable of accurately replicating internal model mismatches and external disturbances commonly present in actual control environments. This allows the system to identify transfer functions that closely approximate real-world behavior. Third, the system can reflect sampling inaccuracies in speed and torque measurements [41]. Finally, compared with full-scale train experiments, the HILSE features lower experimental costs, more accessible test conditions, and reduced safety protection requirements [42]. In the development and production of actual tracking controllers, the HILSE play an indispensable role in evaluating the performance of tracking control algorithms.

**Figure 10:**
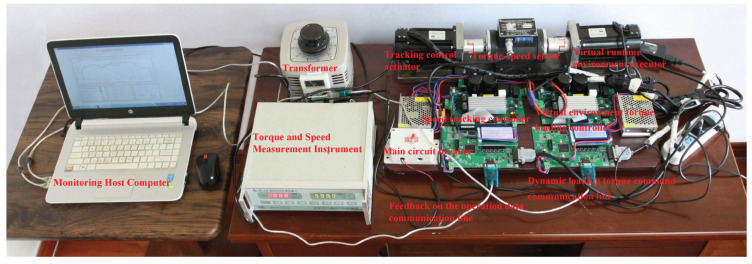
Physical diagram of the HILSE platform for urban rail train tracking control.

As shown in Figure 10, both the speed tracking controller and the torque loading controller for the virtual environment are composed of a 24 V circuit board power supply, a driver circuit board, and a control circuit board that includes a core processing chip and an LCD display. These components are connected to the supervisory host computer via serial communication cables. The host computer is used to receive real-time feedback data from the tracking control operation and to send real-time torque loading commands corresponding to the virtual operating environment. Based on the host computer software interface and the LED display on the control board shown in Figure 10, the current position and speed of the train, as well as their deviations from the target position and speed, can be clearly observed.

The specific configuration of the HILSE used for urban rail train tracking control in this study is described below. The supervisory host computer is equipped with a CPU Core i9-7920X and runs MATLAB version 2016b. Both the speed tracking controller and the torque loading controller for the virtual operating environment are based on the TMS320F28335 core CPU chip. The programs for these controllers were developed using Code Composer Studio (CCS) version 5.5. The model of the LCD display on the control circuit board is 12864B V2.0, and communication is implemented via the RS-485 protocol. The torque and speed sensor, along with its measurement device, is model No. JN338. The sensor features a speed measurement range of 6000 rad/min and a torque measurement range of 20 N·m. Both the tracking control actuator and the virtual environment actuator use permanent magnet synchronous motors (PMSMs) with a rated power of 750 W. These motors have a rated voltage of 220 V, a rated torque of 2.4 N·m, and a peak current of 4.2 A. The operating voltage is 100 V, and the simulated speed-to-distance ratio is 10 rad/min to 1 km/h. The speed tracking control system for the PMSMs employs a triple closed-loop vector control structure. The current loop uses proportional-integral (PI) control, the inner speed loop adopts active disturbance rejection control (ADRC), and the outer torque loop utilizes the MPC introduced in this paper. For comparison purposes, other conventional tracking control algorithms were also implemented under the same framework.

### 5.4. Discussion and Analysis of HILSE Results for Urban Rail Train Tracking Control

**Figure 11:**
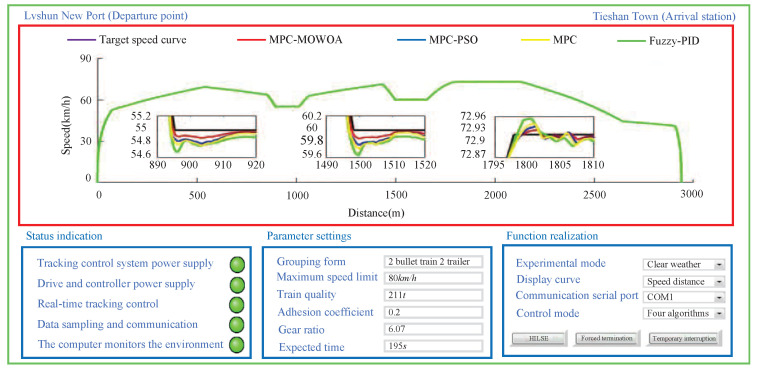
The speed distance curves obtained by different control algorithms.

**Figure 12:**
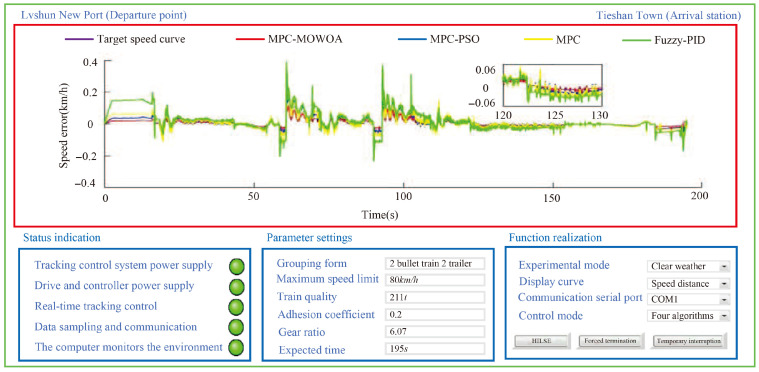
The speed error time curves obtained by different control algorithms.

**Figure 13:**
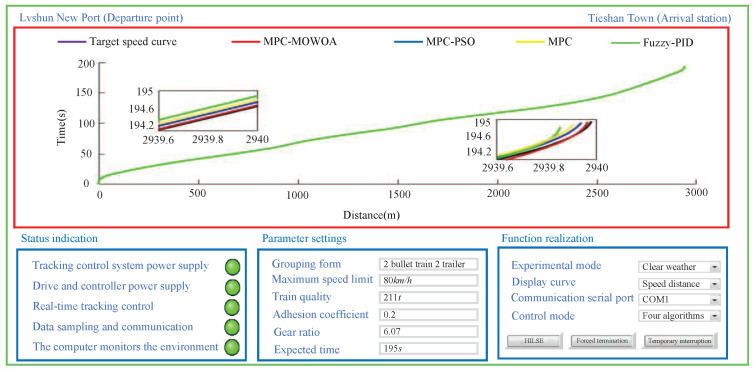
The time distance curves obtained by different control algorithms.

Based on the scenario, to verify the effectiveness of the proposed MPC-MOWOA, the urban rail train tracking control was conducted using three control strategies: MPC optimized by PSO(MPC-PSO) [43], conventional MPC [44], and Fuzzy-PID control [45]. The Fuzzy-PID controller integrates PID control with fuzzy logic, adjusting PID parameters online through fuzzy rules to effectively handle system nonlinearity and disturbances. It was selected as a comparative algorithm because of its widespread application and representativeness in urban rail train speed control, providing a benchmark for evaluating the performance of the proposed predictive control method and highlighting its advantages in constraint handling and speed tracking accuracy. All comparative algorithms were tested under the same simulation environment, train model, operating conditions, and evaluation metrics, and fair and engineering-reasonable comparisons were ensured through appropriate parameter tuning. The simulation results are shown in Figure 11, Figure 12 and Figure 13 and Table 7 and Table 8.

As shown in Figure 11, Figure 12 and Figure 13, during the HILSE, the power supplies for the tracking control system, its driver, and the controller were activated. The real-time tracking control process satisfied the specified tracking constraints, data sampling and communication were smooth, and the host computer enabled real-time supervision of the tracking control process. Compared with other control algorithms used for comparison, the MPC-MOWOA is proposed in this study, which incorporates online adjustment of control parameters, demonstrated superior tracking performance.

As illustrated in Figure 11, Figure 12 and Figure 13, the MPC-MOWOA proposed in this study can adjust control parameters online based on real-time urban rail train tracking conditions, ultimately achieving better overall tracking control performance for urban rail train. The Figure 11 shows that although all four sequentially applied tracking control algorithms enable the train to operate safely and stably throughout the entire route, the proposed MPC-MOWOA method achieves a speed tracking profile that is closer to the target trajectory. It also demonstrates smoother speed response, smaller tracking errors, and effectively avoids the overshoot observed in static MPC. This performance advantage primarily stems from the combination of the online adjustment mechanism of the softening factor and the MPC parameters optimized by MOWOA, enabling the controller to better adapt to load disturbances and dynamic constraint variations caused by track grade changes, thereby maintaining higher trajectory tracking accuracy and control stability under complex operating conditions. The Figure 12 indicates that the proposed algorithm produces a tracking speed curve that better matches the target speed curve, with smaller speed errors at most time points. The Figure 13 demonstrates that the MPC-MOWOA generates a tracking time curve that is more consistent with the target time trajectory, and the actual stopping point is closer to the target stopping point (195 s, 2940 m), indicating better punctuality and precision in stop control performance.

Further analysis of Figure 11, Figure 12 and Figure 13 indicates that the proposed MPC-MOWOA exhibits more pronounced performance advantages in complex operating sections characterized by significant gradient variations and frequent transitions between traction and braking. In these sections, compared with the other control methods, the proposed approach achieves a smoother speed response and smaller tracking errors. This improvement can be attributed to the MPC parameters optimized by MOWOA, which enhance the controller’s ability to cope with load disturbances induced by gradient changes and variations in dynamic constraints, thereby maintaining higher trajectory tracking accuracy and control stability under complex operating conditions.

In Table 8, the $\cos\langle \lambda_{x},\lambda_{T}\rangle$ represents the cosine of the angle between the weight vector $\lambda_{x}$ of the optimal solution *x* and the target weight vector $\lambda_{T}$. Moreover, although ITAE is a single performance index, it can reflect comprehensive system performance.

As shown in Table 7 and Table 8, where the corresponding references of the compared control algorithms are explicitly provided in Table 7 and remain applicable to Table 8 due to identical algorithm configurations, with the Fuzzy-PID adopted as the reference benchmark, the proposed MPC-MOWOA demonstrates clear performance advantages across multiple key evaluation metrics. The results show that MPC-MOWOA achieves an energy consumption reduction of approximately 5.9%, indicating a notable improvement in energy utilization efficiency during train operation. With respect to speed tracking and stopping control accuracy, the time error and distance error are reduced by about 33.2% and 12.4%, respectively, which reflects enhanced tracking precision as well as improved stopping performance. In addition, the comfort index is improved by nearly 7.1%, suggesting that the proposed method effectively suppresses fluctuations during acceleration and deceleration processes, thereby enabling smoother train operation. From the perspective of overall control performance, the ITAE obtained using MPC-MOWOA is reduced by nearly 39.6% compared with that of the Fuzzy-PID, indicating a substantial enhancement in global tracking control quality. Moreover, the proposed approach exhibits improved multi-objective coordination capability, achieving a more balanced trade-off among competing performance objectives than the benchmark method.

Additionally, the simulation results indicate that MPC-MOWOA maintains strong comprehensive performance under complex operating conditions. By conducting coordinated multi-objective optimization of MPC parameters, the proposed method achieves evident improvements in speed tracking accuracy, energy consumption, and comfort. Although practical implementations may be influenced by factors including measurement noise, model uncertainties, and communication or computational delays, which may lead to some performance degradation compared with ideal simulation results, the overall control trends and performance improvement characteristics are expected to remain robust.

Overall, under various comparative criteria such as energy consumption, tracking accuracy, comfort, and comprehensive performance, MPC-MOWOA consistently delivers superior control performance. These results confirm the effectiveness and advantages of the proposed method in addressing the urban rail train tracking control problem, while also highlighting its potential value for practical engineering applications.

## 6. Conclusions

This paper proposes an optimized model predictive control using a multi-objective whale optimization algorithm (MPC-MOWOA) for urban rail train tracking control, with the objective of achieving coordinated optimization among multiple performance criteria, including tracking accuracy, energy consumption, punctuality, stopping precision, and comfort. Within the proposed control framework, an adaptive regulation mechanism based on a fuzzy satisfaction function is introduced to dynamically tune the softening factor in MPC. Meanwhile, the control parameters are adjusted online according to the speed tracking error and its rate of change, thereby effectively enhancing system stability and dynamic adaptability in the presence of model uncertainties, external disturbances, and varying operating conditions.

Considering the problems encountered in MPC parameter tuning, such as strong coupling among parameters, difficulty in manual tuning, and conflicts among multiple performance objectives, a MOWOA is used to optimize the MPC parameters. Furthermore, the algorithm is improved by incorporating a nonlinear convergence mechanism and the Tchebycheff decomposition method, which transforms the original multi-objective optimization problem into a set of scalar subproblems, thereby improving global search capability and convergence stability. Hardware-in-the-loop simulation experiments (HILSEs) conducted on the Lvshun New Port-Tieshan section of Dalian Metro Line 12 demonstrate that the proposed MPC-MOWOA achieves superior performance in terms of ITAE, energy consumption, punctuality, stopping accuracy, and comfort when compared with the benchmark methods. Moreover, the feasibility of this validation approach has been confirmed on an embedded processor (TMS320F28335), overcoming the common limitation in related studies that rely solely on theoretical MATLAB simulations. These results confirm the effectiveness, stability, and practical engineering value of the proposed approach in complex urban rail transit operating environments.

From the perspective of practical engineering applications, the proposed MPC-MOWOA framework is explicitly oriented toward real-world implementation. The multi-objective optimization process is carried out entirely in an offline environment and therefore does not impose any additional computational burden during the online operation of the MPC. Moreover, the proposed approach can be incorporated into existing ATO systems through parameter configuration, without requiring modifications to the core control architecture or the introduction of additional hardware resources. This characteristic ensures good compatibility with current train control systems and supports its practical deployability in engineering applications.

Although the proposed MPC-MOWOA demonstrates satisfactory performance in the conducted simulations, certain limitations still exist and require further investigation.

(I) The experimental validation in this study is currently limited to a hardware-in-the-loop simulation environment (HILSE), and no on-site experiments have been conducted on real urban rail train systems. Although HILSE can reflect the dynamic characteristics of control algorithms in an engineering-oriented environment to a certain extent, its idealized conditions cannot fully capture non-ideal effects encountered in real operation, such as sensor noise, sampling delays, actuator dead zones, and long-term operational disturbances. Consequently, the reliability and robustness of the proposed algorithm in real train operations still require further verification.
(II) The longitudinal train dynamics model adopted in this study is a linear or quasi-linear approximation used for state prediction and control optimization within the MPC framework. This model is obtained through system identification or empirical calibration and can represent the average dynamic behavior under typical operating conditions. However, it may not fully capture extreme nonlinear phenomena, such as wheel slip, wheel spin, or emergency braking scenarios, which constitute a form of model mismatch.
(III) The MOWOA may incur a significantly increased computational burden in high-dimensional objective spaces or large-scale train operation scenarios. Although the scenarios designed in this study satisfy the real-time requirements of the current HILSE platform, real-time control performance may be constrained in more complex or large-scale practical applications.
(IV) In this study, the online adjustment of the softening factor and correction coefficients is based on predefined fuzzy satisfaction indices and velocity error characteristics, without incorporating fully adaptive or learning-based adjustment mechanisms. As a result, when operating conditions vary significantly, the proposed method may exhibit limitations in terms of long-term adaptability and generalization capability.

In view of the limitations discussed above, future research directions can be outlined as follows:

(I) Future studies could consider conducting experimental validation on actual urban rail trains or on more advanced HILSE platforms, in order to systematically evaluate the feasibility, reliability, and long-term stability of the proposed algorithm under real operating conditions. Additionally, upgrading the hardware platform to enhance the performance of embedded processors would provide support for the real-time implementation of more complex control algorithms.
(II) The current linear approximation model may not fully capture extreme nonlinear behaviors, such as wheel slip, spin, or emergency braking. Future research could incorporate nonlinear modeling, online system identification, or adaptive modeling techniques to improve the representation of complex operating conditions, thereby enhancing the robustness of the MPC under model uncertainties.
(III) Future work could explore fast optimization algorithms, distributed computing architectures, or adaptive multi-objective optimization methods to address the computational complexity associated with high-dimensional or large-scale train operation scenarios. Furthermore, integrating urban rail energy efficiency optimization with modern energy systems and intelligent logistics could allow investigation of coordinated operation strategies among train loads, power supply systems, and external energy networks (e.g., hydrogen, electric grid), thereby expanding the practical relevance and application value of the research.
(IV) In the present study, the multi-objective weights are kept fixed during control. Future research could explore dynamic or adaptive weight allocation strategies to achieve more flexible and efficient coordination among multiple performance indices, such as energy consumption, comfort, punctuality, and stopping accuracy, thus further enhancing the overall performance, robustness, and adaptability of the control system under complex operating conditions.

## Figures and Tables

**Table 1:** Comparison of control methods for urban rail train tracking control.

Method	Reference	Advantages	Limitations
Adaptive control	[11]	Parameter adaptation Strong adaptability	Complex implementation Slow convergence
SMC	[12]	High disturbance rejection Strong robustness	Chattering effect Sensitive to control tuning
ILC	[13]	Suitable for repetitive tasks High-precision tracking	Limited effectiveness under non-repetitive conditions Requires many iterations
ADRC	[14]	Strong disturbance rejection Low model dependency	Difficult parameter tuning Theoretical framework not yet fully established
MPC	[15,16]	Handles multiple constraints Strong predictive capability	Computationally intensive High model dependency

**Table 2:** Fuzzy rules of the correction coefficient adjustment quantity.

δ\$\dot{\delta }$	NB	NM	NS	Z	PS	PM	PB
NB	PS	Z	Z	NS	NM	NB	NB
NM	PM	PS	Z	NS	NS	NM	NM
NS	PB	PM	PS	Z	NS	NS	NM
Z	PB	PM	PS	Z	Z	NS	NS
PS	PB	PM	PS	Z	NS	NS	NM
PM	PM	PS	Z	NS	NS	NM	NM
PB	PS	Z	Z	NS	NM	NB	NB

**Table 3:** The primary parameters for urban rail train tracking control.

Parameter Name (Unit)	Parameter Value
Maximum speed limit (km/h)	78
Maximum safety speed limit (km/h)	75
Distance between stations (m)	2940
Expected running time (s)	195
Maximum allowable distance error (m)	±0.2
Maximum allowable error of time (s)	±0.5

**Table 4:** The idealized operational results of the scenario in this study.

Parameter Name (Unit)	Parameter Value
Traction energy consumption (kJ)	72,044
Time error (s)	0.043
Distance error (m)	0.022
Comfort level (m/$s^{2}$/km)	5.044

**Table 5:** The key performance indicators range of the scenario in this study.

Parameter Name (Unit)	Parameter Symbol	Parameter Range
Energy consumption (kJ)	*E*	[70,000, 90,000]
Comfort level (m/$s^{2}$/km)	$K_{Jerk1}$	[5, 9]
Tracking control comfort (m/$s^{2}$/km)	$K_{Jerk2}$	[27, 49]
Distance error (m)	Δs	[0, 0.2]
Time error (s)	Δt	[0, 0.5]

**Table 6:** The optimal control parameters are obtained through MOWOA optimization.

Parameter Name	Parameter Symbol	Value or Range
Softening factor	$\left[\alpha_{\min},\alpha_{\max}\right]$	[0.83, 0.94]
Relaxation factor trend control coefficient	bα	1.59
Initial correction coefficient	$\alpha h_{i}$	0.72
Quantization factor for speed error	$k_{in\_e}$	$\frac{0.5}{3}$
Quantization factor for speed error rate	$k_{in\_\dot{e}}$	$\frac{3}{1.6}$
Adjustment ratio factor for correction coefficient	$k_{out\_\Delta \alpha h}$	$\frac{0.14}{3}$

**Table 7:** Tracking control results of each optimization objectives obtained by different control algorithms.

Control Algorithm	Energy Consumption (kJ)	Time Error (s)	Distance Error (m)	Comfort Level (m/$s^{2}$/km)
MPC-MOWOA	76,423	0.189	0.162	36.927
MPC-PSO [42]	78,543	0.227	0.170	37.844
MPC [43]	79,423	0.243	0.179	38.263
Fuzzy-PID [44]	81,254	0.283	0.185	39.751

**Table 8:** Comprehensive performance indexes about tracking control quality obtained by different control algorithms.

Control Algorithm	$\cos\langle \lambda_{x},\lambda_{T}\rangle$	Train Speed ITAE
MPC-MOWOA	0.9732	504
MPC-PSO	0.9671	587
MPC	0.9640	712
Fuzzy-PID	0.9566	835

## Data Availability

The original contributions presented in this study are included in the article. Further inquiries can be directed to the corresponding author.

## Abbreviations

The following abbreviations are used in this manuscript:

ATO	automatic train operation
MPC	model predictive control
DMC	dynamic matrix control
WOA	whale optimization algorithm
MOWOA	multi-objective whale optimization algorithm
ITAE	integral of time-weighted absolute error
HILSE	hardware-in-the-loop simulation experiment
SMC	sliding mode control
ILC	iterative learning control
ADRC	active disturbance rejection control
